# Cardiac Geometry and Function in Patients with Reflex Syncope

**DOI:** 10.3390/jcm13226852

**Published:** 2024-11-14

**Authors:** Giorgia Coseriu, Patricia Schiop-Tentea, Csilla-Andrea Apetrei, Iulia-Georgiana Mindreanu, Adriana-Daniela Sarb, Madalina-Patricia Moldovan, Roxana Daiana Lazar, Teodora Avram, Roxana Chiorescu, Gabriel Gusetu, Sorin Pop, Edwin Kevin Heist, Dan Blendea

**Affiliations:** 1 Faculty of Medicine, University of Medicine and Pharmacy Iuliu Hatieganu, 400012 Cluj-Napoca, Romania; coseriugiorgia@gmail.com (G.C.); dblendea1@gmail.com (D.B.); 2 Niculae Stancioiu Heart Institute, 400001 Cluj-Napoca, Romania; 3 Emergency County Hospital, 400347 Cluj-Napoca, Romania; 4 Rehabilitation Hospital, 400066 Cluj-Napoca, Romania; 5 Massachusetts General Hospital, Boston, MA 02114, USA; 6 Harvard Medical School, Cambridge, MA 02115, USA

**Keywords:** reflex syncope, ventricular theory, left ventricle dimension and function, small left atria, predictive model

## Abstract

Reflex syncope (RS) is the most prevalent form of syncope, yet its pathophysiology and clinical presentation are not well understood. Despite controversy, the ‘ventricular theory’ remains the most plausible hypothesis to explain RS in susceptible patients. Certain assumptions regarding the geometry and function of the heart are essential in supporting this theory. Given these considerations, the goal of this review was to try to integrate data on heart morphology and function in a phenotype of a patient susceptible to RS. Previous research suggests that a small left ventricle and atria, in addition to a normo- or hypercontractile myocardium, predispose to more syncopal events. These findings have been confirmed in different subsets of patients, including those with small heart and chronic fatigue syndrome, highlighting common pathophysiologic pathways in these subgroups of population. Heart geometry and function seem to play a role in different treatment strategies for RS patients, including the administration of medications, pacing, and possibly cardioneural ablation. In addition, parameters related to the geometry of the heart chambers and of the electrical activation of the heart seem to have predictive value for syncope recurrence. These parameters could be included in the future and improve the accuracy of predictive models for RS.

## 1. Introduction

Current estimates suggest that 50% of the global population will experience at least one syncopal episode in their lifetime [1]. Syncope affects men and women in roughly equal proportions [2] and follows a tri-modal age distribution, with incidence peaks occurring in the third, seventh and ninth decades of life [3]. Studies indicate that up to 5% of individuals experiencing syncope require evaluation in the emergency department, with a hospitalization rate of 40%, resulting in an annual economic impact of approximately USD 2.4 billion [4]. In the absence of a standardized diagnostic approach, a significant proportion of patients presenting with syncope receive suboptimal treatment [5]. Among the different types of syncope, the vasovagal one is the most common in routine clinical practice [6].

Despite its significant incidence, syncope remains poorly understood. One of the leading theories explaining the mechanism of reflex syncope (RS) is the ventricular theory, which highlights the role of a small yet forceful contraction of the left ventricle (LV) in mediating the reflex arc in susceptible individuals. However, while this theory is widely accepted, it is also controversial, as the implications of cardiac geometric and functional aspects have not been thoroughly examined. 

This review aims to provide a comprehensive perspective on the pathophysiology of RS, emphasizing the significance of cardiac dimensions and functional implications. We discuss treatment strategies, outline the limitations of current knowledge, and explore future perspectives related to predictive models for syncopal recurrence.

## 2. RS—From Definition to Pathophysiology

Syncope is a condition defined by transient loss of consciousness, loss of postural tone and spontaneously reversible symptoms [7]. Having more substrates, syncope is subdivided as follows: RS, or neurally mediated syncope, syncope due to orthostatic hypotension and cardiac syncope [8,9].

Depending on the trigger type, RS can be subsequently classified into four entities, as follows: vasovagal syncope (VVS), situational syncope, carotid sinus syndrome and non-classical syncope [1].

### 2.1. Pathophysiological Components of RS

Brain perfusion is strongly related to blood pressure (BP), which in turn is dependent on peripheral resistance (PR) and cardiac output (CO) [10]. Because of its need for almost 20% of the CO, the cerebral tissue is equipped with a limited intrinsic capacity to regulate its blood supply during hemodynamic variations. However, when the autoregulation mechanism is no longer able to compensate the fluctuations in hemodynamic parameters, brain perfusion decreases, causing syncope [11,12,13,14].

### 2.2. Triggers and Receptors

Under certain circumstances, people with susceptibility to RS tend to have an impaired cardiovascular reflex, consisting of arterial hypotension and bradycardia, with subsequent loss of consciousness [15,16,17].

RS is often related to orthostatic stress, and standing could be a trigger for syncope in these patients. Emotional distress, coughing [18], sneezing, swallowing and micturition [1] are also frequent triggers for RS in susceptible people. These triggers act by stimulating receptors with various locations, including the brain, blood vessels, as well as the respiratory, digestive and urinary tracts.

**Figure 1:**
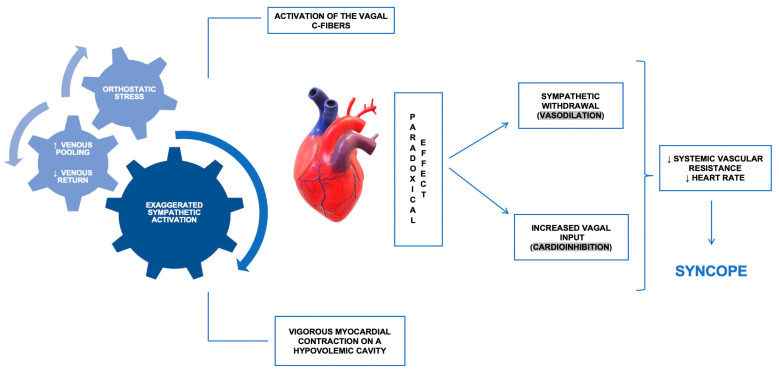
The Bezold–Jarisch reflex as a consequence of orthostatic stress (a concept in the pathophysiology of the vasovagal syncope): orthostatic stress leads to a redistribution of the circulatory volume towards the inferior extremities of the body (‘venous pooling’), causing a decrease in venous return. The exacerbation of sympathetic tone will induce a vigorous myocardial contraction on an empty left ventricle, with further stimulation of cardiac mechanoreceptors. The C fibers’ activation can lead to a paradoxical effect, resulting in an exaggerated vagal tone, sympathetic withdrawal and syncope. ↑—increased, ↓—decreased.

The importance of the cardiac mechanoreceptors in mediating the vasovagal reflex in patients with RS has been highlighted in the “ventricular hypothesis”, which states that venous pooling induced by prolonged orthostatism enhances the sympathetic tone, which further stimulates the cardiac mechanoreceptors through a forceful ventricular contraction on a hypovolemic cavity (Figure 1). All these events lead to the activation of the afferent pathway of the reflex arc, ultimately resulting in syncope [10,12].

### 2.3. The Afferent Pathway and the Integration Center

The signals received from the peripheral receptors or from the central nervous system directly are transmitted to the nucleus solitarius via the vagal and glossopharyngeal nerves. The nucleus solitarius has connections with the dorsal and ambiguous nuclei, the spinal cord, the hypothalamus and the brainstem [15].

### 2.4. The Efferent Pathway and the Types of Response

After processing the information, the cerebral nuclei send impulses to the effectors via the efferent pathway, promoting a decrease in BP, which finally leads to a loss of consciousness [19].

As BP is dependent on CO and PR, there are three mechanisms that explain clinical manifestations in patients with RS [19]. These types of response are identified based on the hemodynamic behavior to tilt-table testing (TTT). The *cardioinhibitory response* explains hypotension through sinus bradycardia or sinus pauses caused by an exaggerated vagal influence on the heart. The *vasodepressor response*, characterized by hypotension as well, appears as a consequence of a diminished sympathetic tone, leading to secondary vasodilation [19]. However, in clinical practice, syncope is often based on a *mixed type of response*, meaning that there is a variable cardioinhibitory and vasodepressor component [17,19,20].

### 2.5. Age-Related Changes in RS Hemodynamics

According to a recent study, the hemodynamic changes in RS are influenced by age, with younger subjects having higher heart rates and a more attenuated increase in systemic vascular resistance when compared to elderly patients [21].

### 2.6. RS as Part of the Evolutionary Progress

Some researchers propose that VVS may have played a significant adaptive role throughout human evolution. Bracha et al. [22] highlighted that during major world conflicts, a common cause of death was injuries from sharp objects. Individuals with a phobia of sharp objects often experienced syncope when exposed to such stimuli, even before an attack. This fainting response could mimic death, potentially discouraging the aggressor and allowing the victim to escape unharmed [23]. In contrast, Diehl et al. suggested that VVS acts as a protective mechanism for mammals experiencing acute hemorrhage [23]. The arterial hypotension associated with syncope may help reduce blood loss and promote clot formation [23]. Given these survival benefits, VVS can be viewed as an essential component of the human instinct for self-preservation.

## 3. Heart Geometry and Function in Patients with RS

The autonomic nervous system intricately regulates heart rate and vascular tone, so dysfunction within this system can lead to abnormal ventricular responses. For instance, vagal stimulation may induce bradycardia and decrease myocardial contractility, both of which impact blood flow and BP. While central nervous system mechanisms, such as baroreceptor reflexes, are often emphasized in understanding RS, the ‘ventricular theory’ highlights the role of autonomic imbalance in directly affecting heart function during syncope episodes [24].

The ventricular hypothesis was described in 1956, when Sharpey–Schafer [25] hypothesized that the receptors and afferents (also known as *the C fibers*) of the vasovagal reflex are located in the LV and that a forceful myocardial contraction on an underfilled heart chamber (induced by orthostatic venous pooling) could mediate the autonomic reflex with further syncope. This is a form of the *Bezold–Jarisch reflex* [23,26,27,28]. Despite ongoing advancements, the ventricular hypothesis remains a contentious topic, as it fails to account for several clinical and experimental observations [18,29,30]. The discrepancies in findings may stem from limitations in current research, including small sample sizes, a lack of standardized methodologies and the limited accuracy of echocardiographic measurements. Nevertheless, several recent studies have provided data that support the ventricular theory, as outlined in the table that summarizes several syncope studies (Table 1). Although studies suggest a role for cardiac geometry in the pathogenesis of RS, no experimental data currently exist to thoroughly evaluate the impact of cardiac geometric parameters on the underlying mechanisms of RS.

### 3.1. Exploring Geometry and Function of the LV in Patients with RS

It is known that RS occurs mostly in the upper position [9]. Consequently, TTT has become a common tool in studies evaluating cardiac geometry and function in these patients (Table 1) [31,32,33]. However, data derived from research utilizing TTT to induce syncope must be interpreted with caution, as TTT represents an artificial method of triggering syncope that may not accurately reflect the mechanisms involved in spontaneous events.

**Table 1:** Studies evaluating cardiac geometry and function in patients with RS.

Authors	Study Groups	Echocardiographic Parameters	Results
SHALEV et al. [34]	Group I (control; n = 11) Group II (− TTT; n = 4) Group III (+ TTT at baseline; n = 9) Group IV (+ TTT with Isoproterenol; n = 5)	LVED, LVES areas and diameters LV FS	LVES and LVED areas are significantly smaller in patients with + TTT (*p* < 0.02; *p* < 0.03) LV FS is significantly higher in patients with + TTT (*p* < 0.001)
MIZUMAKI et al. [31]	Group I (+ TTT at baseline; n = 13) Group II (+ TTT with Isoproterenol; n = 14) Group III (control; n = 20)	LVED, LVES diameters LV FS	Patients with + baseline TTT had significantly smaller LVED and LVES diameters compared with those with + TTT after Isoproterenol infusion or controls (*p* < 0.01, *p* < 0.005) No change in FS LV was observed in those with + baseline TTT (*p* = NS)
LEE et al. [32]	Group 1 (− TTT after Isoproterenol; n = 5) Group 2 (+ TTT after Isoproterenol; n = 16) Group 3 (+ baseline TTT; n = 5)	LVED, LVES diameters LV FS	No significant changes were observed between the three groups regarding LVEDD or LVESD during TTT (*p* = NS) LV FS in patients with + TTT at baseline increased significantly during TTT (*p* < 0.0001)
LIU et al. [35]	Group I (− TTT; n = 11) Group II (+ TTT; n = 10)	LVED, LVES indexed volumes LVED, LVES diameters SV Mid wall shortening Stress-corrected endocardial FS Meridional and circumferential ESS Segmental LV wall thickness	At rest: no significant differences between patients with positive (+) TTT and patients with negative (−) TTT regarding LVED and LVES volumes, SV, endocardial FS, mid-wall shortening LVED volume index, segmental wall thickness or mid-wall shortening (*p* = NS) Significantly important decreases in LVEDVI, SV, stress-corrected FS, stress- corrected mid-wall shortening, cESS and mESS in patients with + TTT (*p* < 0.05)
YAMANOUCHI et al. [36]	Group I (+ TTT; n = 7) Group II (control; n = 9)	LVED, LVES indexed volumes SV LVEF	SV and LVEF decreased significantly during TTT in patients with + TTT (*p* = 0.02; *p* = 0.001) A faster rate of LVEDV reduction during orthostatism in patients with + TTT when compared to controls (*p* = 0.04)
KOZLOVSKI et al. [37]	Group I (+ TTT; n = 7) Group II (− TTT; n = 32)	LVES, LVED volumes LVEF LV posterior wall slope	No significant changes in LVES and LVED volumes or LVEF during TTT between the + TTT and − TTT patients (*p* = NS) Tendency for higher values of LV posterior wall slope in patients with + TTT (*p* > 0.05)
FOLINO et al. [38]	Group I (+ TTT; n = 15) Group II (− TTT; n = 8)	Sw, Aw—anterior and inferior LV wall LAA, LAV LVEF LVEDV	Aw (inferior and anterior wall) decreased significantly in patients with + TTT (*p* < 0.005) Sw (inferior wall) increased significantly in patients with + TTT (*p* < 0.05) Sw (anterior wall) increased significantly in patients with + TTT (*p* < 0.05) All patients had similar reductions in the LAA and LAV; LVEF did not show significant variation between the 2 study groups during TTT; LVEDV decreased similarly in the 2 groups during TTT
GOEL et al. [39]	Group A (patients with + TTT and co-morbid conditions; n = 30) Group B (patients with + TTT and no co-morbid conditions; n = 30) Group C (patients with − TTT; n = 22) Group D (control; n = 20)	LVED, LVES diameters LVEF RVSP (right ventricular systolic pressure) Radial, circumferential and longitudinal strain of the LV	LV septum and LV posterior wall were significantly thicker in patients with positive TTT and co-morbid conditions compared to patients with positive TTT and no co-morbid conditions (*p* < 0.05) LV end-diastolic diameter was significantly greater in patients with positive response to TTT compared with controls (*p* < 0.05) Global longitudinal strain was greater in patients with positive response to TTT compared to patients with negative TTT response (*p* < 0.05)
MOON et al. [40]	Group I (patients with + TTT; n = 152) Group II (patients with − TTT; n = 82)	LVED, LVES diameters LVEF LV mass LAVI LAAPD/height Early mitral inflow peak velocity (E), late mitral inflow peak velocity (A) Diastolic septal mitral annular velocity (E′) and peak systolic mitral annular velocity (S′)	LVED diameter was significantly smaller and LVH was more common in patients with − TTT (*p* = 0.015, *p* = 0.027) LAVI and the ratio of LAAPD to height were significantly smaller in + TTT group compared to the patients with − TTT (*p* < 0.001) E′ was higher in + TTT group when compared with − TTT group (*p* = 0.004)
BASANALA et al. [41]	Group I (patients with + TTT; n = 45) Group II (patients with − TTT; n = 40)	LVED volume LAAPD LAV and LAVI LAEF Early mitral inflow peak velocity (E), late mitral inflow peak velocity (A), E/A ratio Peak systolic velocity (S′), early diastolic velocity (E′) and late diastolic velocity (A′)	LAV was significantly smaller in patients with + TTT compared with patients from − TTT group (*p* = 0.03) Significantly higher E/A ratio in the + TTT group (*p* = 0.02) Patients with + TTT response had moderately lower LAVI and LAEF values compared to patients with − TTT response (*p* = 0.05)
BLENDEA et al. [42]	Group I (patients with recurrence of syncope; n = 60) Group II (patients without recurrence of syncope; n = 145	LVED, LVES diameters	LVED and LVES diameters were significantly smaller in patients with recurrence of syncope *(p* < 0.001; *p* = 0.009)
BLENDEA et al. [43]	Group I (patients with + response to TTT; n = 91) Group II (patients with − response to TTT; n = 125)	LVED, LVES diameters LVES and LVES diameters indexed to height	LVES and LVED diameters indexed to height were significantly smaller in patients with positive TTT compared to patients with negative TTT (*p* = 0.04)

Abbreviations: TTT—tilt-table test, + TTT—positive TTT, − TTT—negative TTT, LVES—left ventricular end-systolic, LVED—left ventricular end-diastolic, LV FS—left ventricular fractional shortening, SV—stroke volume, LVEDV—end-diastolic volume, LVEDVI—left ventricular end-diastolic volume index, ESS—end-systolic stress, LAA—left atrial area, LAV—left atrial volume, LAVI—left atrium volume index, LAAPD—left atrial antero-posterior diameter, LAEF—left atrium ejection fraction.

The first relatively large study to assess heart’s geometry and function by echocardiography while performing TTT in patients with previous unexplained syncope was performed by Shalev et al. [34]. Based on their findings, the authors reinforced the idea that patients with positive TTT tend to have a significantly smaller LV and a more vigorous myocardial contraction when compared to controls or patients with negative response to TTT [34]. Even if the results were in agreement with the ventricular theory, this study had several limitations, including a heterogeneous study population, a non-standardized protocol concerning image acquisition [36] and limited accuracy in defining ventricular dimensions [34].

Another study that included patients that underwent TTT was performed by Mitzumaki et al. and did not find any significant difference in heart geometry and function between patients with RS induced by TTT alone and those who additionally required Isoproterenol infusion [31]. Secondary to the echocardiographic study, it was concluded that patients with a positive baseline TTT had an important decrease in the LV dimensions right before the syncopal event and that a similar effect might also be seen in non-susceptible patients after Isoproterenol infusion. On the other hand, contrary to observations made by Shalev et al. [34], the contractility of the LV did not significantly increase in patients with a positive baseline TTT. A limitation of this study was related to a suboptimal quality of the echocardiographic images that were used to assess cardiac geometry and function [31].

A study by Lee and colleagues, which analyzed patients undergoing TTT, revealed that patients who developed RS had a more vigorous myocardial contraction compared to patients with a negative TTT, but no significant difference was found in LV cavity size [32]. The results suggested that RS was the result of a beta-adrenergic hypersensitive status with consequent hypercontractility of the LV during orthostatism, and that a preexisting empty cavity was not necessarily a prerequisite for syncope [32]. The somewhat discordant observations concerning LV diameter variations during RS were mainly attributed to a change in the study protocol, namely to the correction of dehydration and hypovolemia by fluid supply right before performing the TTT. Another limitation was related to the suboptimal quality of echocardiographic images, which might have led to errors in measurements [32].

Liu et al. showed that using the LV fractional shortening to describe heart contractility in patients with RS may not be as helpful as thought because of its load dependency. In contrast to previous studies that used fractional shortening, they estimated the LV systolic function by quantifying the myocardial wall stress, and the results showed that patients with TTT-induced RS, apart from having a smaller LV, tend to have a reduction in myocardial contractility [35]. The authors concluded that the activation of the C fibers from the LV in patients with RS was most probably related to multiple triggers and not only to a forceful myocardial contraction. One limitation of their study was represented by a suboptimal quality of the echocardiographic images. It was also suggested that an increase in the LV contractility might have occurred right before syncope, without the possibility to record these changes by echocardiography [35].

Another study that revealed a relationship between a small LV size and RS was conducted by Yamanouchi et al. [36]. They revealed a fast reduction in the LV end-diastolic volume during TTT as an essential trigger for syncope in subjects with RS. According to their observations, this subcategory of patients had a greater venous capacitance compared to the controls, with a more significant reduction in ventricular preload during orthostatism [36]. Other findings were related to a significant reduction in the ejection fraction and stroke index in subjects with tilt-induced RS [36]. Even though not entirely in agreement with the ventricular theory, it is important to note that the study group was small, and that larger similar studies need to be performed in order to validate the outcomes.

In a study conducted by Kozlowski et al., there was a reduction in LV volumes during TTT; however, no difference was observed between the TTT-positive and the TTT-negative patients [37]. One important limitation of this research was represented by the small number of patients.

Folino and colleagues evaluated ventricular contractility in patients with RS using tissue-Doppler imaging (TDI) at the level of the anterior and inferior wall of the left ventricle, minutes after performing TTT. In contrast to other similar studies, observations showed that patients with RS, despite a significant sympathetic activity, do not exhibit a marked increase in ventricular contractility [38]. The results suggest that the role of myocardial hypercontractility in mediating the vasovagal reflex is limited, and that there are other factors involved in triggering the reflex. The results also revealed that patients with a positive TTT seem to have a more important reduction in terms of left atrial contractility, questioning the idea of a possible relationship between atrial contraction, decrease in CO and syncope. It is worth noting that this study revealed a significant drop in left atrial and LV end-diastolic volumes during TTT, as well. One important limitation of the study was related to the impossibility to assess the echocardiographic parameters continuously during TTT, so a possible hypercontractile phase could have been missed [38].

Another research study that aimed to define the LV function in patients with RS was conducted by Goel et al. [39], who used speckle tracking echocardiography to define myocardial contraction. The study showed that, compared with the control subjects, the endocardial longitudinal strain in patients with positive TTT was significantly lower (*p* <0.05). Interestingly, the thickness of the ventricular septum and posterior LV wall, as well as the left atrial volume index, were smaller in the positive TTT when compared with controls, although the results did not reach statistical significance [39]. One major limitation of the study, similar to the most studies described above, was a relatively small number of patients.

Current research appears to confirm that smaller LV end-systolic and end-diastolic dimensions in RS patients are associated with a positive TTT response and syncope recurrence [42,43].

Comorbid conditions, such as hypertension and diabetes, can alter cardiac geometry and may therefore act as potential confounders in studies examining the role of cardiac geometry in syncope pathogenesis. However, in the studies we reviewed, where data were available, no significant differences in the prevalence of these comorbidities were observed between study groups. This suggests that any potential confounding effects likely did not meaningfully impact the study results. Several other potential confounding factors may have influenced the current findings. It is also important to consider that the patients’ hydration status and body size could have affected the intracardiac volumes. Different studies have addressed these potential confounders to varying degrees, and their effects may not have been completely controlled for.

The analyzed studies exhibited several limitations, primarily concerning the accuracy of the assessment of geometrical and functional echocardiographic parameters. As a result, the clinical applicability of the findings remains uncertain. Moreover, to accurately validate the implications of heart geometry and function in patients with RS, it is essential to conduct studies involving larger patient cohorts.

### 3.2. From Syncope to Small Heart and Chronic Fatigue Syndrome

A small heart shadow on the chest roentgenogram in people with persistent and disabling fatigue characterizes a separate entity called the *small heart syndrome (SHS)* [44]. This condition was first described in 1826 by Laennec, who noted that patients with smaller hearts experienced persistent fatigue, dyspnea, palpitations and syncope because their heart is unable to fulfill body needs [45]. In 1944, Master highlighted once again the correlation between reduced heart dimensions on the chest roentgenogram and symptoms, justifying the clinical presentation in these patients as a consequence of a decrease in venous return and CO [45]. These and several other observations [44,45] suggest that the presence of SHS is correlated with a higher incidence of orthostatic syncope. One could wonder if there is at least a partial overlap between RS and SHS.

Findings in SHS can be extrapolated to the role of the geometry of the heart in the pathophysiology of the RS. Therefore, it was speculated that a smaller diameter of the left chambers of the heart, along with an excessive LV contraction, may play an important role in mediating the autonomic reflex in patients with RS [34]. Even if there are some studies sustaining this theory, there is still controversy regarding whether changes in heart geometry and function are indispensable elements in the pathophysiological process of the RS [31,32,35,36,46].

Another important remark made by Master et al. concerned the similarities between symptoms described by patients with SHS and those of patients with chronic fatigue syndrome (CFS) [47]. CFS is defined as a persistent, relapsing and disabling fatigue observed mainly in young people, often accompanied by other nonspecific symptoms, such as headache, sleep disorders, malaise, concentration difficulties or musculoskeletal pain [44].

**Figure 2:**
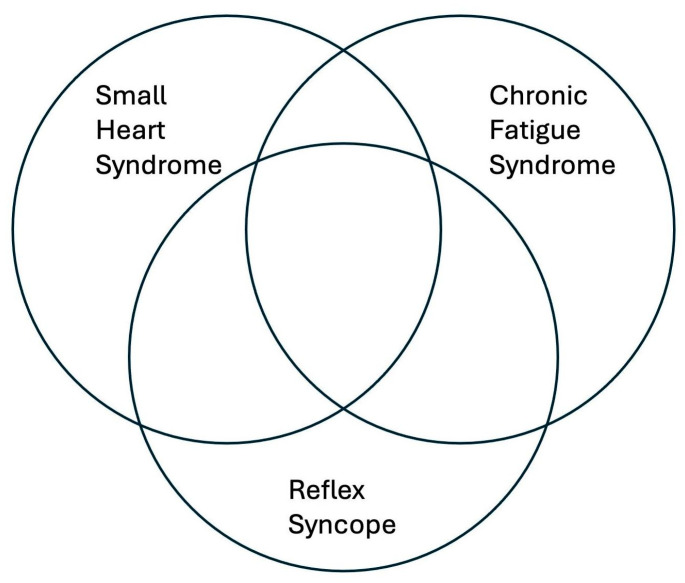
Small heart syndrome, reflex syncope and chronic fatigue syndrome are entities that overlap partially and often have several clinical features in common.

A study conducted by Miwa et al. showed that an increased proportion of Japanese patients with CFS also associate with SHS [45]. Based on the echocardiographic analysis, subjects with CFS and concomitant SHS had significantly smaller LV chamber dimensions and significantly smaller LV stroke volume and CO when compared to controls, even after indexing the parameters to the body surface area [45]. These observations highlighted the fact that a small heart, which is also a feature of patients with RS, plays an important role in the genesis of CFS [45]. This may suggest a partial overlap between the three entities (Figure 2).

In an attempt to better define the relationship between patients with CFS and patients with SHS, Miwa et al. performed another study, which included 42 young patients diagnosed with CFS, 62% of whom were further diagnosed with concomitant SHS [44]. Once again, the echocardiographic analysis showed the presence of significantly smaller LV chamber sizes, smaller stroke volume and cardiac index in patients with CFS and associated SHS compared to controls and to those with CFS and normal heart dimensions. Moreover, repeated echocardiographic evaluation highlighted the existence of a significant improvement in all geometrical and functional parameters during remission phase (defined as symptom improvement, without meeting the criteria for CFS) in patients with CFS and associated SHS. These results suggest the existence of a strong correlation between the severity of CFS and cardiac function. As CFS triggers were mostly associated with dehydration and consequent decrease in preload, it is now thought that cardiac impairment in subjects with CFS and SHS during the exacerbation phase is responsible for symptom aggravation [44].

Even if the general interest to study people with small hearts has not been very high, there is, however, proof that this phenotype interconnects several medical conditions, so understanding the geometry and function of the heart in these patients may help to better define the relations between a small heart, symptomatology and treatment options.

## 4. Left Atrium Geometry and Function in Patients with RS

Even if attention has been mostly directed towards the connection between geometrical and functional aspects of the LV and syncope, there are studies suggesting that the left atrium (LA) also plays a major role in the pathogenesis of RS. Given the fact that the LA systole is responsible of 20% of the diastolic left ventricular filling, one can imagine that changes in LA mechanics and volume have consequences on the CO, especially during orthostatism [41].

The importance of LA dimensions in patients with syncope was highlighted by Folino et al., who identified a decrease in the LA dimensions minutes after performing TTT in both positive and negative responders [38]. Furthermore, observations showed that during orthostatism, subjects with RS tend to have a decrease in atrial contraction despite important sympathetic activation. Based on these findings, it was hypothesized that RS might be the consequence of a decreased CO, as a less efficient atrial contraction during diastole contributes to an improper filling of the LV during orthostatism [38].

Moon et al. [40] highlighted the relation between LA volume variations and RS by showing that a small LA indexed volume (LAVI) is an independent predictor of RS during TTT, whereas a LAVI greater or equal to 36 mL/m^2^ was not associated with fainting during orthostatic stress. Another finding of the study was that the subjects with smaller LAVI experienced syncope earlier than the controls. Furthermore, it was also shown that the anteroposterior diameter of the LA was smaller in patients with positive TTT [40].

Using the LA ejection fraction as a marker of function, Basanalan et al. demonstrated that atrial contractility was diminished in patients with positive TTT when compared to those with negative TTT [41]. Additionally, the LA volume was found to be significantly smaller in those who experienced RS, findings which were in accordance with those previously revealed by Moon et al. Therefore, their study reinforced the idea that orthostatic-induced RS might be a consequence of not only reduced ventricular filling but also decreased atrial contractility [41].

## 5. Treatment Strategies and Heart Geometry

Despite RS having usually a benign course, frequent syncopal recurrences represent an important source of mental stress, with serious repercussions on the quality of life [48]. According to recent estimations, the recurrence rate in people with RS is about 25 to 30% [24].

Cardiac geometry and function play an important role in the pathogenesis of the RS, and they also need to be taken into consideration when applying different treatment methods in these patients.

### 5.1. Increasing Intravascular and Intracardiac Volumes

The first and most recommended therapeutic approach in these patients is represented by the non-pharmacological strategy, mostly related to good hydration status maintenance, increased salt intake, lifestyle changes, trigger avoidance, isometric counterpressure maneuvers and tilt training sessions [9].

However, estimations suggest that 14% of patients with RS remain symptomatic despite the non-pharmacological methods, so that additional treatment may be required [9].

It has been shown that Fludrocortisone could help reduce syncopal recurrence by increasing intravascular volume. Although *Prevention of Syncope Trial 2* did not meet its primary objective of demonstrating that Fludrocortisone was able to reduce the likelihood of VVS by the specified risk reduction of 40%, it demonstrated a significant effect after dose stabilization, with significant findings in post hoc multivariable and on-treatment analyses [49].

### 5.2. From Ventricular Theory to Pacing and Ablation Therapy

The interest in pacing in VVS has increased after data suggesting the efficacy of this procedure in reducing syncopal burden when applied in selected patients. Published in 2012, the *ISSUE-3* double-blind, randomized placebo-controlled trial was the first study to demonstrate a 32% absolute and a 57% relative risk reduction in syncopal events in subjects with recurrent syncope and a significant cardioinhibitory response, who benefited from dual-chamber pacing with rate-drop response versus sensing alone [6].

One pacing modality that uses changes in cardiac geometry and function in patients with VVS is the closed-loop stimulation (CLS). Developed by Biotronik in the late 1980s, the CLS system represents a pacing algorithm that uses right ventricular impedance variations during cardiac cycle as a surrogate for myocardial contractility [6,50]. Depending on the content (blood or tissue) surrounding the pacing electrode from the right ventricle, there is a continuous oscillation of impedance during systole and diastole. Thus, when the ventricular cavity is full of blood, impedance decreases, whereas during systole, as the cavity empties, impedance increases [50]. In patients with cardioinhibitory VVS, the CO decreases, mainly due to either bradycardia or asystole. Consequently, in order to prevent syncope, there is a rise in myocardial contractility, leading to changes in impedance and pacing activation [51,52].

Over the past years, CLS pacing has become a therapy with proven efficacy in patients with refractory VVS and a predominantly cardioinhibitory component [53]. Moreover, the results from the *SPAIN trial* showed that, apart from preventing syncopal events, dual-chamber CLS (DDD-CLS) pacing had also significantly improved the quality of life in these subjects, compared to those using the DDI simple mode [52,53].

With a class I indication and level A of evidence, pacing therapy is now recommended in patients above the age of 40 with recurrent or unpredictable RS, severe clinical presentation, and ECG evidence for cardioinhibition during carotid sinus massage, TTT or implantable cardiac monitoring [53].

Despite a good success rate, in up to 25% of the stimulated patients there was a syncopal relapse, mainly due to a coexistent vasodepressor response [53]. This observation is based on the results of a meta-analysis from 2018, where it was shown that the estimated 3-year recurrence rate of VVS was significantly higher in patients with a positive TTT versus those with a negative TTT (33% versus 2%) [54]. Since a positive response to TTT was the only significant predictor for syncopal reoccurrence, it is now evident that in this subcategory of patients, a specific therapy to counteract the hypotensive susceptibility is needed besides cardiac pacing [53].

Given the mentioned limited efficacy of conventional treatment methods in preventing recurrences of RS, another therapeutic strategy has been proposed [55,56]. Cardioneuroablation (CAN) targets the main pathophysiological substrate of the VVS, which is the excessive parasympathetic influence on the heart. With good results in treating vagal-mediated bradycardia and also some, albeit limited, effects in preventing associated vasodilation, CAN has been mostly studied in patients with cardioinhibitory and mixed VVS [16,57]. This is different from cardiac pacing, which is only efficient in patients with cardioinhibitory phenotype. Technically, the modulation of the intrinsic autonomic nervous system by CAN is induced by performing a selective endocardial denervation in the major ganglionated plexi, located next to the left and the right atria [29,58,59].

Even though vagal denervation has been validated in several studies as an efficient method with encouraging results concerning syncopal burden reduction, more analysis is needed in order to allow its use in routine clinical practice. From our knowledge, the echocardiographic parameters reflecting heart geometry and function have not yet been specifically included in a predictive model concerning the success rate of CAN in patients with VVS. It is conceivable that the presence of a small heart, mainly associated with the vasodepressor or mixed phenotype, might play a role in patient selection for CAN. However, there is still a need for research on this subject in the future.

### 5.3. Beta-Adrenergic Receptor Blockers

The ventricular theory posits that a key pathophysiological factor in RS is an elevated adrenergic tone, which stimulates the cardiac mechanoreceptors in the LV. This theory prompted the hypothesis that beta-adrenergic blockers might help mitigate the receptors activation, by lowering the sympathetic tone and reducing the inotropic effects [60]. However, two randomized trials investigating atenolol and propranolol for VVS prevention did not demonstrate significant efficacy in reducing syncope episodes in the studied populations [60,61]. Likewise, a study by Sheldon et al. reported no significant benefits of metoprolol in reducing VVS recurrence. While beta-blockers are generally not recommended for VVS, they may still be beneficial in selected patients, where elevated sympathetic tone plays a pronounced role in pathogenesis.

## 6. Summary and Future Directions

**Figure 3:**
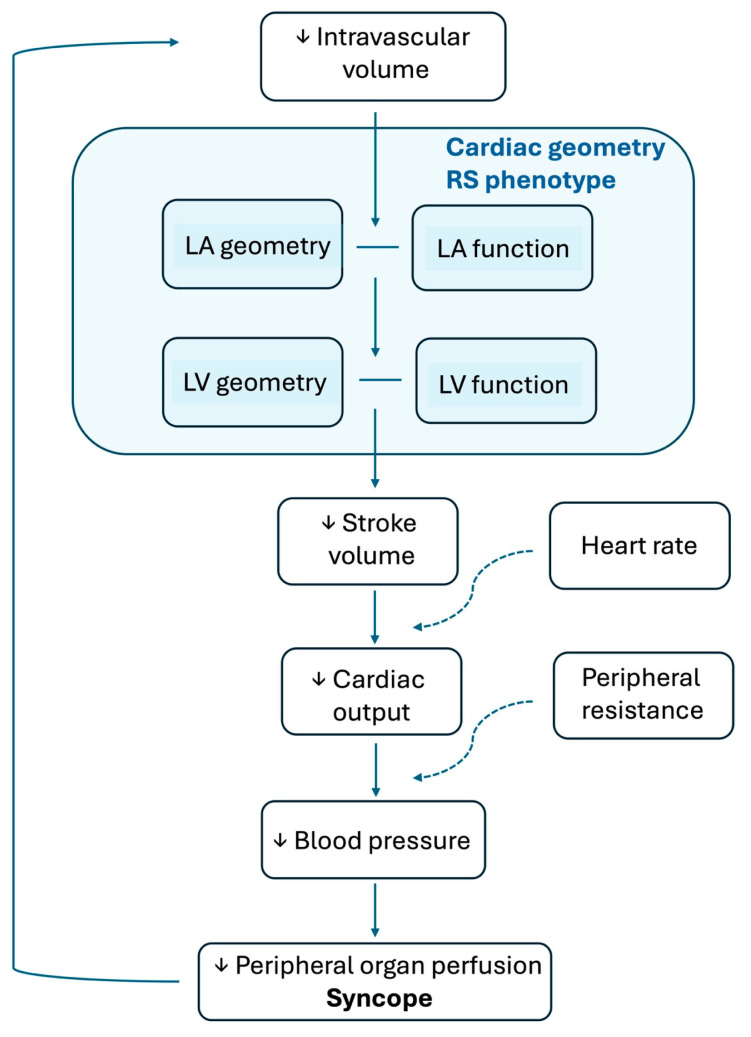
The cardiac geometry reflex syncope phenotype is characterized by a small-sized left heart and variable function, which facilitate the onset and progression of a syncopal episode. RS = reflex syncope, LA = left atrium, LV = left ventricle, ↓ = decreased.

**Figure 4:**
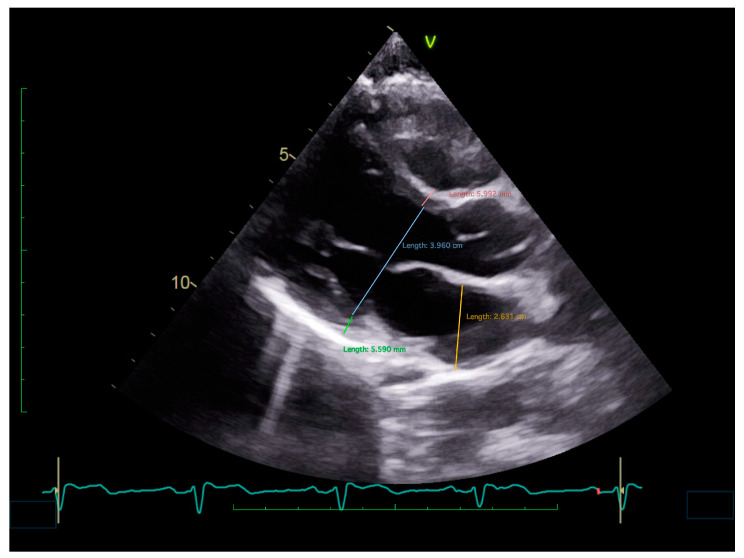
An ultrasound image of a parasternal long-axis view of the heart, showing small left cavities and thin ventricular walls in a patient with reflex syncope.

Based on the data accumulated in the literature, a phenotype of the cardiac structure in patients with RS is emerging (Figure 3). These patients seem to have smaller, normo- or hypercontractile ventricles, as well as smaller atria (Figure 4) [42]. This resembles, to a certain extent, the ventricular phenotype of patients with hypertrophic obstructive cardiomyopathy, in whom a small LV cavity and a normo- or hypercontractile ventricle seem to participate in the pathogenesis of syncope. In patients with hypertrophic cardiomyopathy, however, the ventricular wall thickness is increased, as it is the internal diameter of the LA. Although the evidence is limited, a small LV size may have predictive value for recurrent syncope, warranting further research in this area.

There are several knowledge gaps in the evaluation and management of patients with syncope, all presenting potential opportunities for future research.

Increasing low intracardiac volumes appears to be a logical approach, given their significance in the pathogenesis of RS. While volume repletion and increased salt intake have been suggested as potential interventions, their efficacy remains unproven. Counterpressure maneuvers and midodrine have demonstrated some effectiveness in relatively small studies; however, robust evidence from large, randomized controlled trials is still lacking. It remains to be determined in future research whether certain subgroups of patients with RS would derive greater benefits from these therapies.

There is no one-size-fits-all strategy for treating RS. Understanding the cardiac geometry, which correlates with tendencies toward vasodepressor or cardioinhibitory mechanisms, may aid in individualizing treatment. Currently, pacing and CAN are recommended for patients with cardioinhibitory and mixed phenotypes but are not indicated for vasodepressor syncope [62,63]. Patients exhibiting vasodepressor responses may respond more favorably to interventions such as fluid resuscitation and midodrine. Phenotypes characterized by low adenosine [64] and low norepinephrine levels [65], as well as syncope associated with mast cell activation syndrome [66,67], often display predominant vasodepressor mechanisms. These patients may benefit from volume repletion, increased salt intake, and alpha agonists, alongside more targeted therapies such as theophylline, reboxetine, sibutramine, antihistamines and mast cell stabilizers [68].

RS is a transient, unpredictable and most often relatively infrequent phenomenon. Given this, the ideal preventive therapy should be one that is administered only when needed, based on continuous monitoring of the risk of syncopal recurrence. Monitoring BP, the electrocardiogram, the intracardiac volume and contractility by using sensors similar to CLS pacing or to the pulmonary pressure sensor monitoring system in heart failure [69] might be useful in guiding a therapy administered on an as-needed basis (similar to Flecainide in atrial fibrillation). Isolated very low voltage QRS in frontal ECG leads and flat QRS loops on the frontal vectorcardiogram correlate with small LV end-diastolic diameter and low intravascular volume and have been demonstrated to predict recurrent RS [42,70]. One could envision a monitoring device that could record these electrocardiographic parameters in addition to BP, pulse and intracardiac pressure and could be used to continuously assess the risk of syncope recurrence. Making this type of electronic monitoring system accessible to the general population could greatly simplify the management of patients with RS. This approach would not only enhance their quality of life, but also help reduce hospitalization costs associated with recurrent syncopal episodes. While personalized treatment has the potential to significantly enhance patients’ quality of life, its clinical adoption may face several barriers, particularly regarding the methodology of implementation and the associated economic costs.
